# Supplementary material for: An idiosyncratic zonated stroma encapsulates desmoplastic liver metastases and originates from injured liver
Source: Nat Commun. 2023 Aug 18;14:5024. doi: 10.1038/s41467-023-40688-x (PMC10439160; doi:10.1038/s41467-023-40688-x)
Supplement: Supplementary file 1 — Supplementary Information [file 41467_2023_40688_MOESM1_ESM.pdf]

An idiosyncratic zonated stroma encapsulates desmoplastic liver metastases and originates from injured liver

Carlos Fernández Moro, Natalie Geyer, Sara Harrizi, Yousra Hamidi, Sara Söderqvist, Danyil Kuznyecov, Evelina Tidholm Qvist, Media Salmonson Schaad, Laura Hermann, Amanda Lindberg, Rainer L. Heuchel, Alfonso Martín-Bernabé, Soniya Dhanjal, Anna C. Navis, Christina Villard, Andrea C. del Valle, Lorand Bozóky, Ernesto Sparrelid, Luc Dirix, Carina Strell, Arne Östman, Bernhard Schmierer, Peter B. Vermeulen, Jennie Engstrand, Béla Bozóky, Marco Gerling

Supplementary Figures 1-11
Supplementary Tables 1-3

Supplementary Figure 1

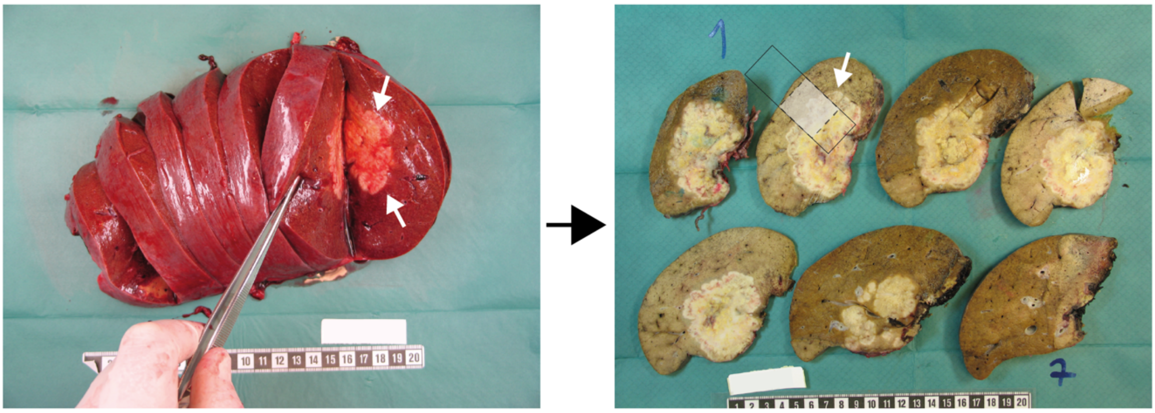

Supplementary Figure 1: Macroscopic sampling of liver metastases. Related to Figure 1b. Macroscopic images of a liver resection specimen. Left panel: Processing of the fresh specimen for initial slicing for adequate formalin fixation and identification of all metastases (arrows). Right panel: For routine diagnostics, after formalin fixation, the liver is cut into approximately 0.5 cm thick slices that are assessed macroscopically to identify all metastases and to select tissue for paraffin embedding. These macroscopic images were used to identify the largest diameter of each metastasis for extended annotations. The arrow indicates the slice with the largest diameter, the size of a routine histology glass (black lines) is shown in the image, and the grey area with dashed lines indicates the size of a standard cassette for histological embedding. The tape measure on the pictures indicates centimeters.

Supplementary Figure 2

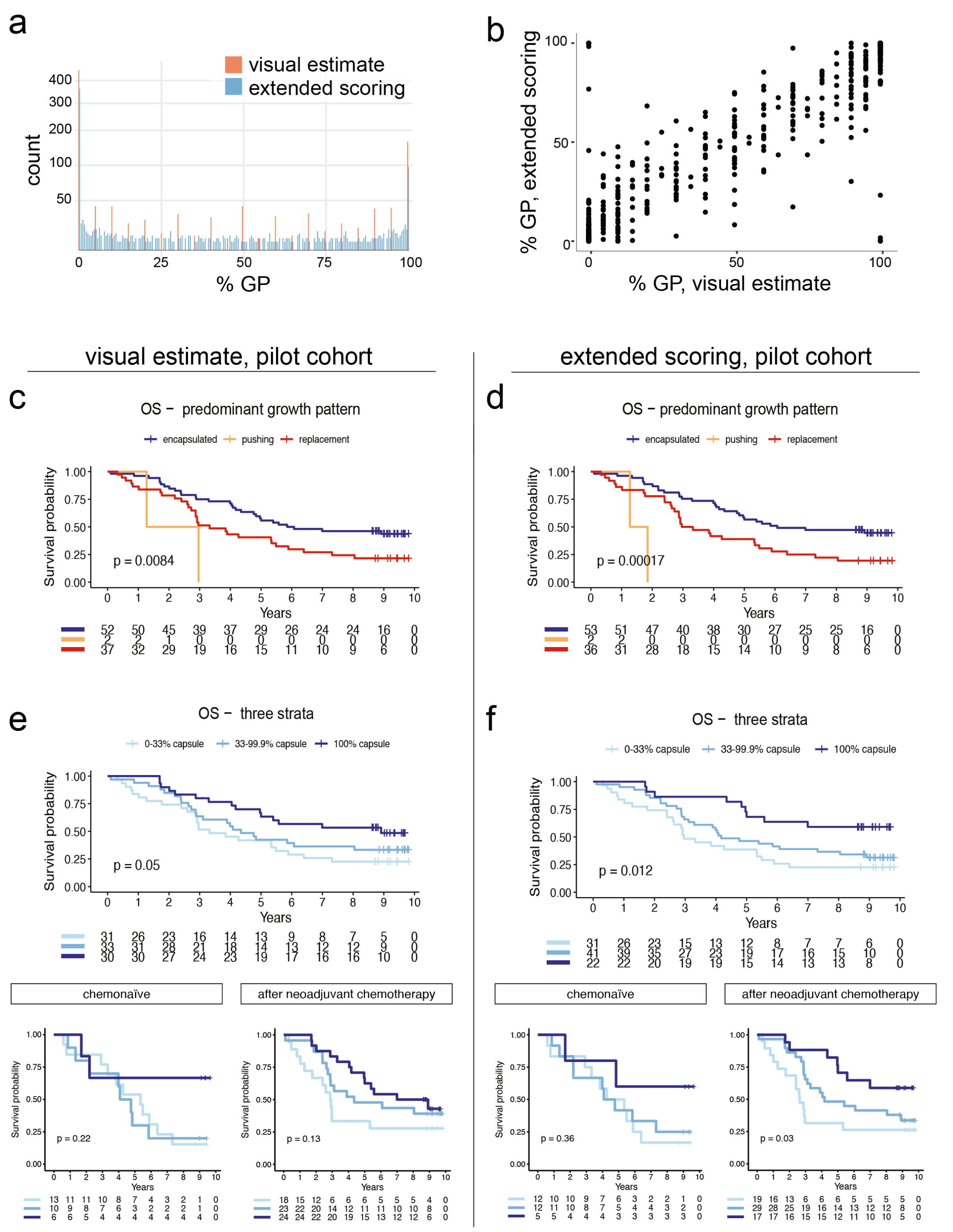

Supplementary Figure 2: Visual estimates and extended scorings - distribution and prognostic value. a) Distribution of the results of visual estimates *vs*. digital annotations per slide (as percentage of any growth pattern per slide). **b)** Scatter plot of visual (horizontal) and extended (vertical) scorings of all growth patterns by the same raters (combined scores of two raters in [a] and [b]). **c – f)** Overall survival (OS) for the same patients when using visual scoring ([c] and [e]) or extended scoring ([d] and [f]) with the indicated stratifications. Log-rank p-values (two-sided) are given in the plots. Note that (c) and (d) show data from n = 91 patients, rather than *n* = 94 because *n* = 3 had growth pattern fractions of 50% for two patterns and hence could not be assigned a "predominant" pattern.

Supplementary Figure 3

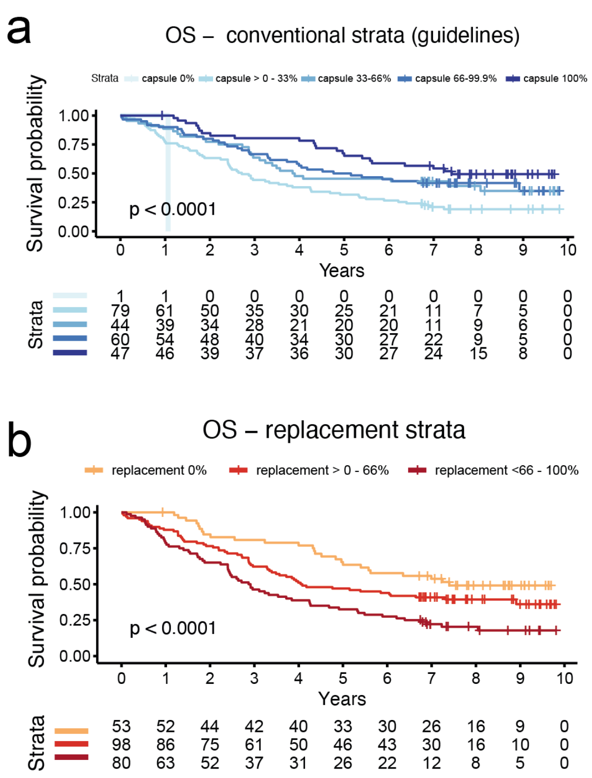

Supplementary Figure 3: Stratification of overall survival according to the growth patterns. a) Overall survival (OS) for previously tested strata of the degree of encapsulation. **b)** OS for the indicated strata of replacement-type growth. Log-rank p-values (two-sided) are given in the plots. OS – conventional strata, p = 0.0000579; OS – replacement strata, p = 0.0000965.

Supplementary Figure 4

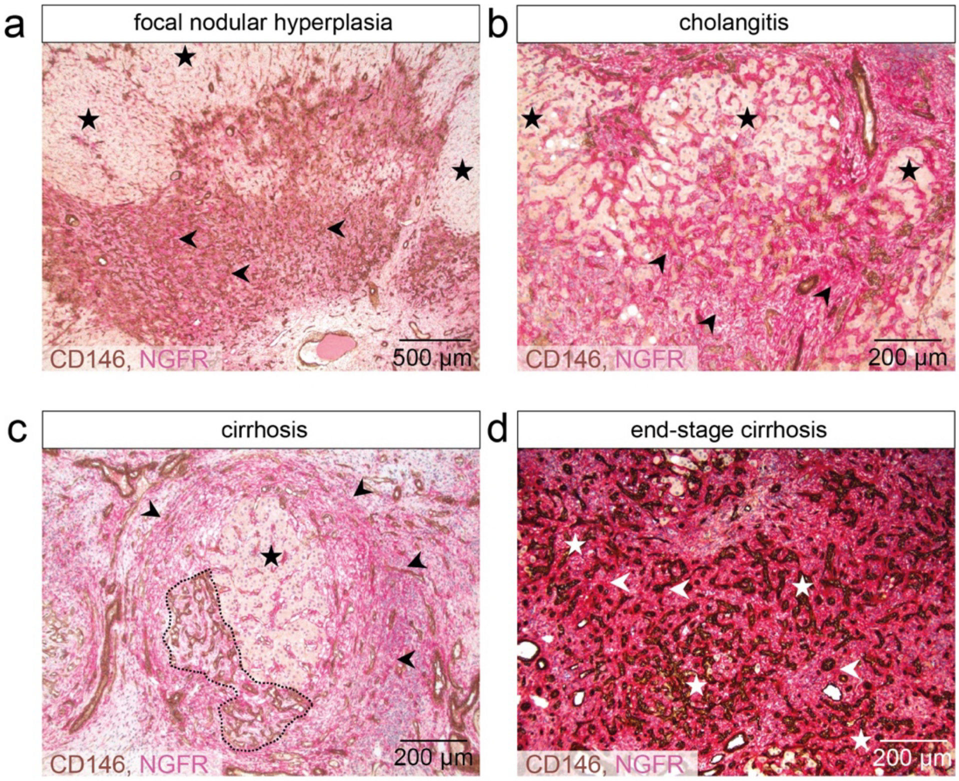

Supplementary Figure 4: Stromal phenotype in benign fibrotic liver conditions. Staining for nerve growth factor receptor (NGFR, stroma, red) and cluster of differentiation 146 (CD146, ductular reaction, brown) are shown for the indicated diseases: **a) Focal nodular hyperplasia (FNH).** Hepatocytes are delimited by fibrous tissue composed of $NGFR^{+}$ stroma, containing prominent ductular reaction ($CD146^{+}$). Stars: hepatocytes; arrowheads: ductular reaction; representative of n = 2 FNH patients. **b) Cholangitis**. Hepatocytes surround the inflamed portal triad and show atrophy with concomitant development of $NGFR^{+}$ fibrosis. Stars: hepatocytes; arrowheads: fibrous stroma; representative of n = 2 patients with cholangitis. **c) Liver cirrhosis**. Hepatic tissue shows partial atrophy, a rim of $NGFR^{+}$ stroma surrounds remnants of hepatocyte nodules, and ductular reaction ($CD146^{+}$) has developed. Star: remnant of hepatic nodule; arrowheads: fibrous rim. Dotted region: ductular reaction. **d) Alcoholic steatohepatitis,** associated with advanced liver cirrhosis with intensive ductular reaction ($CD146^{+}$) embedded in a strongly $NGFR^{+}$ fibrous stroma. Stars: ductular reaction, arrowheads: fibrous stroma. Panels (c) and (d) are representative of n = 7 patients with cirrhosis.

Supplementary Figure 5

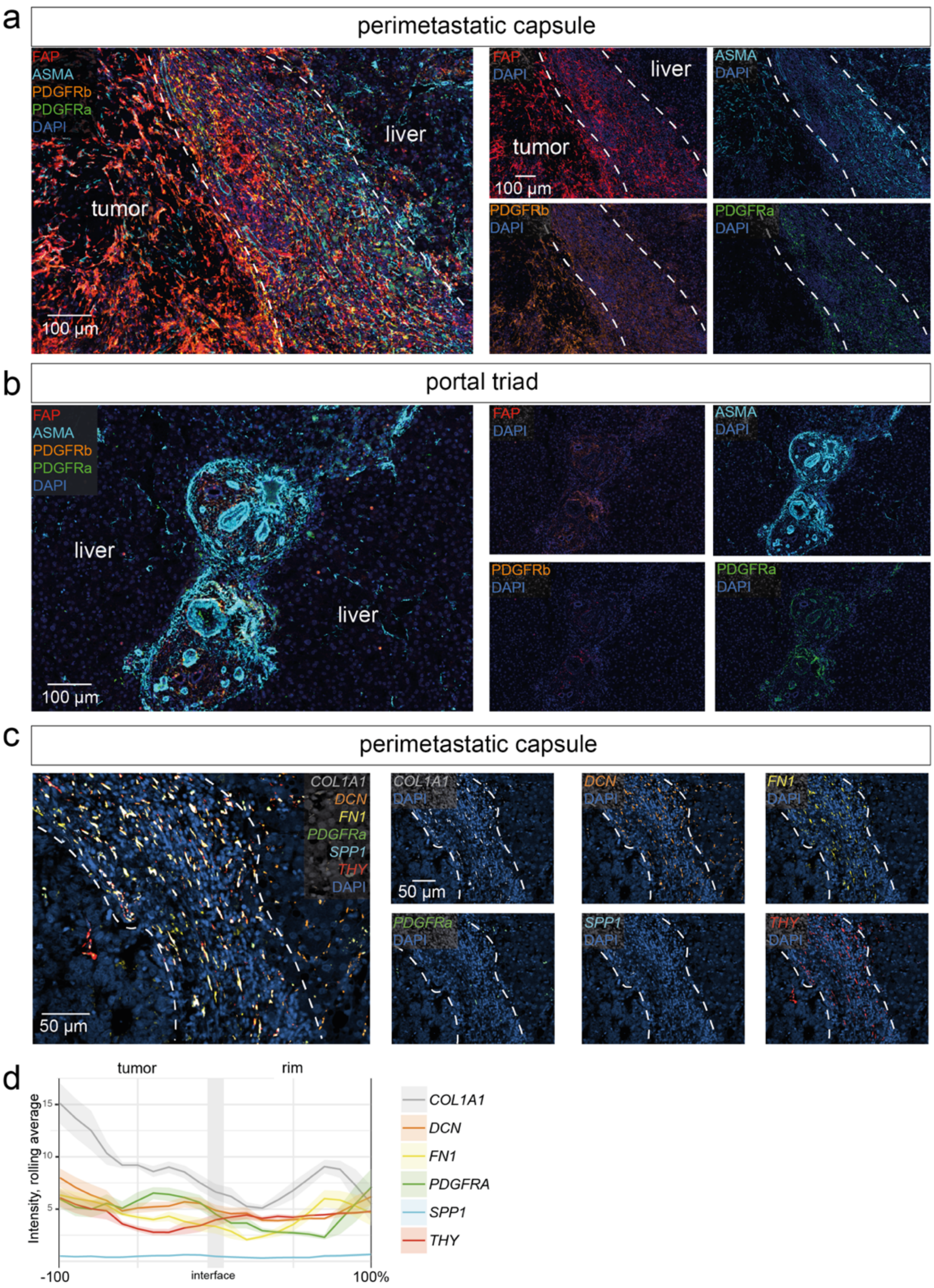

Supplementary Figure 5: Multiplex imaging of the fibrotic rim. a) – b) The same metastasis as in Figure 3d-f is shown. **a**) High magnification of a representative multiplex-immunofluorescence stain (of n = 6 metastases) for the indicated proteins in the capsule, dotted lines indicate the inner and outer capsule border. The right panels show the single channels; note the opposing gradients of fibroblast-associated protein (FAP) and alpha smooth-muscle actin (ASMA). **b)** Larger portal triad devoid of tumor. Scanning settings are the same as in (a). The portal triad is positive for ASMA and largely negative for FAP. For (a) and (b), autofluorescence subtraction was used for the channels ASMA and PDGFRa due to artifacts in the liver parenchyma at green wavelengths; thresholding has been used for those two channels with similar settings for (a) and (b). **c)** Representative image of multiplexed RNA in situ hybridization with probes for the indicated transcripts, using 4′,6-diamidino-2-phenylindole (DAPI) for nuclear staining (representative of n = 8 regions from n = 2 metastases). Large panel to the left shows composite (collagen a1a [*COL1A1*], decorin [*DCN*], fibronectin-1 [*FN1*], platelet-derived growth factor receptor alpha [*PDGFRA*], osteopontin [*SPP1*], Thy-1 cell surface antigen [*THY*]), right panel shows single channels. Single channels were adjusted independently by adjusting min/max display values to remove background (autofluorescence in the liver parenchyma) and ease visualization of signals. **d)** Quantification of stain abundance of the different transcripts across the tumor center and rim. The lines represent rolling averages, the areas represent 95% confidence intervals (n = 8 regions of interest from n = 2 metastases).

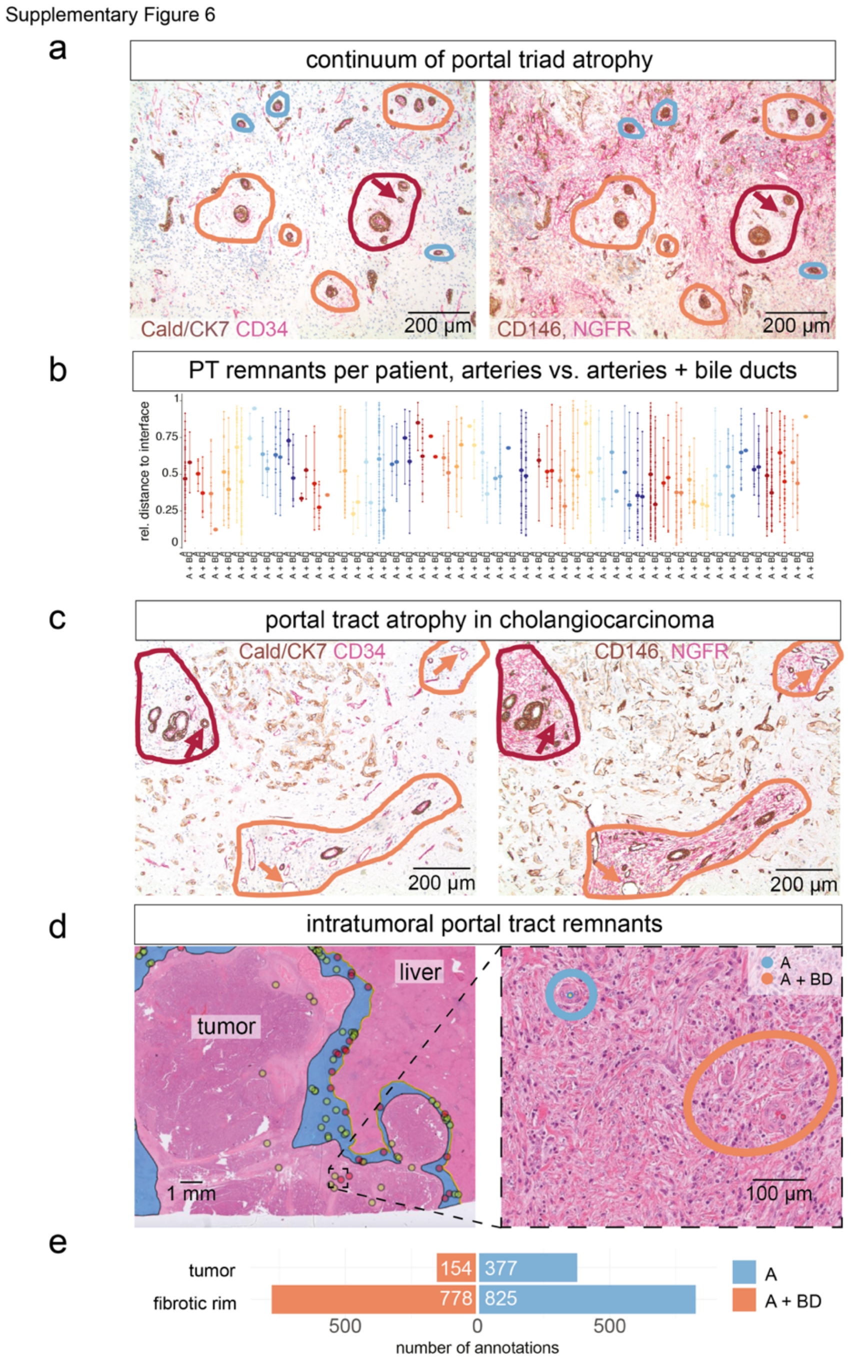

Supplementary Figure 6: Quantification of liver parenchymal remnants. a) Different degrees of portal triad (PT) atrophy in colorectal cancer liver metastases (CLRM) are indicated by different colors: Red: scant portal triads with preserved branches of the hepatic artery, bile duct (arrows), and variably portal stroma. Orange: remnants of portal triads with preserved branches of the hepatic artery and variably portal stroma. Blue: most atrophic remnants, where only branches of the hepatic artery remain. Representative of $n > 5$ routinely stained. metastases. **b)** A and A+BD remnants per patient, different colors denote different patients (note, similar colors are used for different patients). Mean (large dot), individual datapoints and minimum to maximum range (whiskers) are shown. **c)** Portal triad (PT) remnants in a cholangiocarcinoma. Red: scant porta zones with preserved branches of the hepatic artery, bile duct (arrows), and variably portal stroma. Orange: remnants of porta zones with preserved branches of the hepatic artery and variably portal stroma. Remodeled branches of vein can also be detected (orange arrows). Representative of n = 9 patients with cholangiocarcinoma. **d)** Whole-slide image (WSI) annotated for the rim and PTs: blue area marks the rim; red dots mark PTs consisting of artery and bile duct (A+BD), green dots mark PTs consisting of artery remnants (A). Magnification in the right panel, A remnants highlighted in blue, A+BD remnants highlighted in orange. Representative of $n = 49$ metastases. **e)** Quantification of the distribution of intratumoral PT remnants compared to the PT remnants in the rim; absolute numbers are given, without correction for the area. $n =$ 49 patients, $n = 141$ WSIs. Cald: Caldesmon. CK7: Cytokeratin 7, CD34: Cluster of differentiation 34.

Supplementary Figure 7

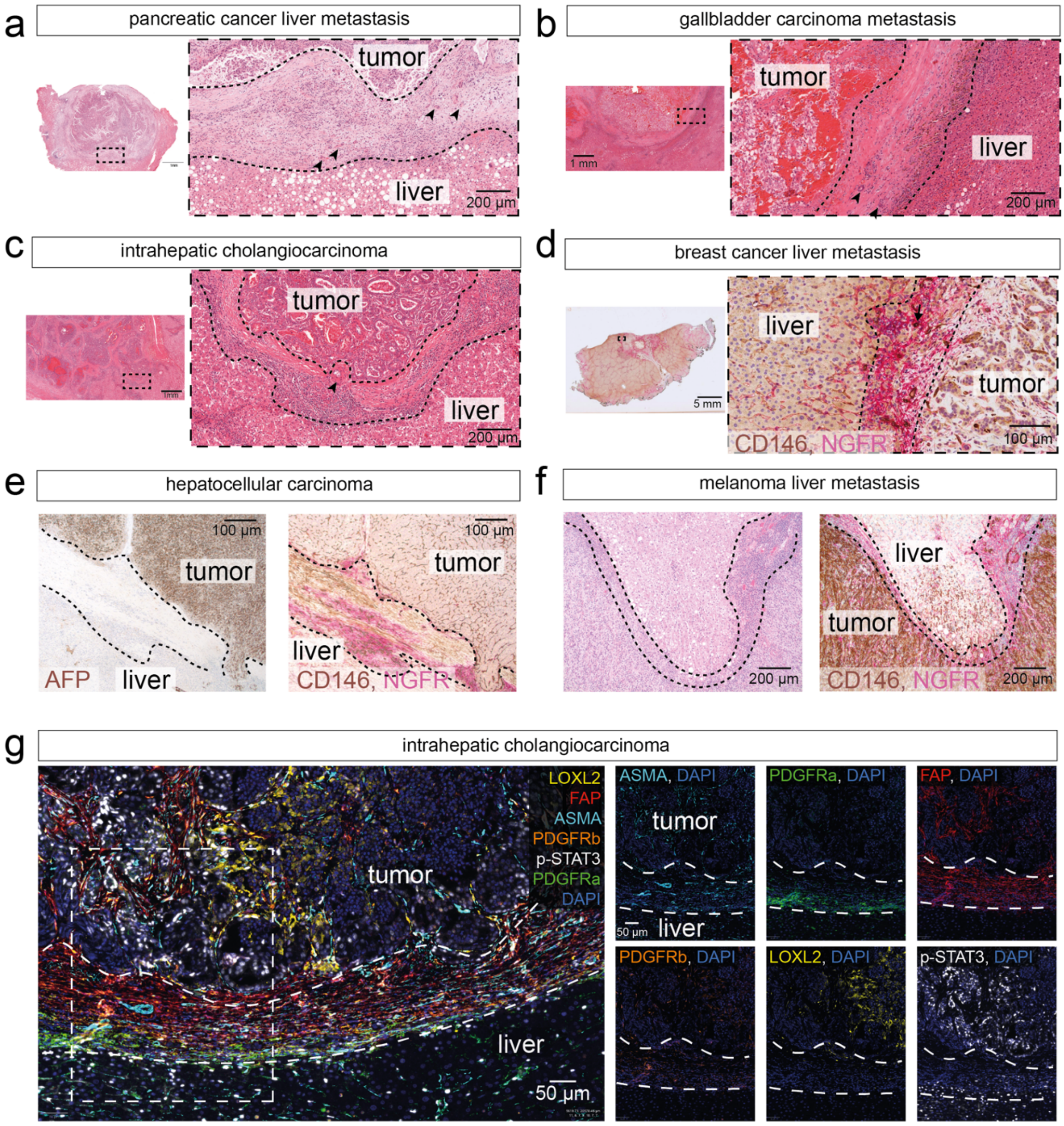

Supplementary Figure 7: Morphology of the perimetastatic capsule in non-colorectal cancer liver metastases. a-c) Hematoxylin and eosin (H&E) stains of liver metastases of pancreatic (a) and gallbladder (b) cancer, and intrahepatic cholangiocarcinoma (c). Arrowheads indicate portal tract remnants. Representative of metastases/tumors from n = 4 patients (pancreatic cancer), n = 4 patients (gallbladder cancer), n = 9 patients (intrahepatic cholangiocarcinoma). **d-e)** Duplex immunohistochemistry for cluster of differentiation 146 (CD146) and nerve growth factor receptor (NGFR) in the indicated tumors. Representative for n = 3 (breast cancer, d), n = 3 (hepatocellular carcinoma, e), and n = 3 (melanoma, f). In (e), the tumor is identified with alpha-fetoprotein (AFP, left panel). Note that the melanoma cancer cells in (f) are positive for CD146 (which is also known as melanoma cell adhesion molecule, MCAM). The left panel in (f) shows a H&E stain. The arrow in (d) points at a $CD146^{+}$ portal tract remnant in the capsule. **g)** Multiplex immunofluorescence for the indicated proteins (corresponding to the panel shown in Figure 3d-f). The dashed lines indicate the inner and outer borders of the peritumoral capsule. Representative of n = 3 cholangiocarcinomas. Thresholding was used for the channels depicting ASMA and PDGFRa to remove background in the liver parenchyma at green wavelengths.

Supplementary Figure 8

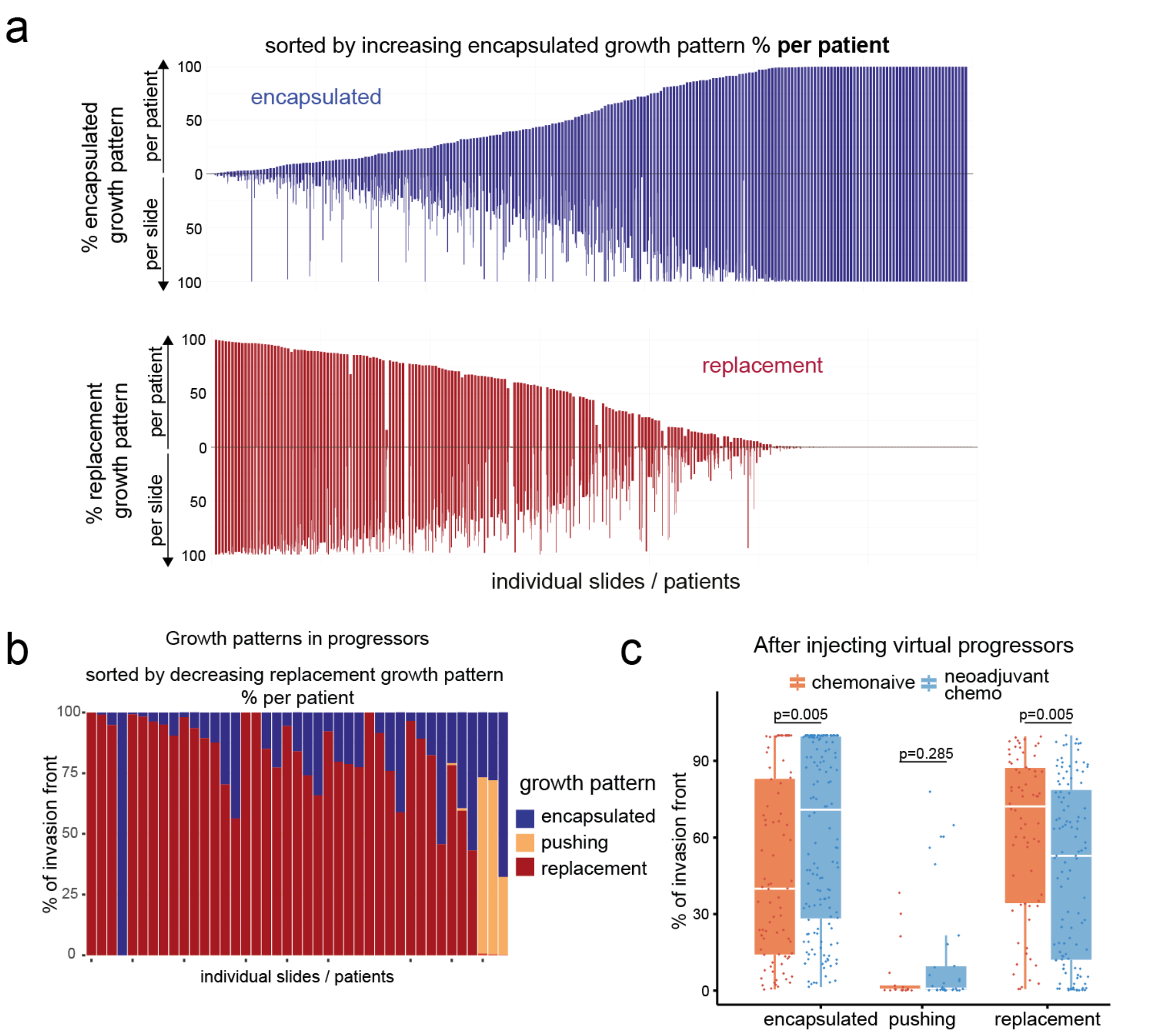

Supplementary Figure 8: Growth patterns distribution per slide and effect of chemotherapy. a) The upper panel shows the percentages of the encapsulated pattern per assessed total length of the tumor-liver interface. The bars above the x-axis show the percentage per individual patient. The bars below the x-axis show the percentages per individual slide, corresponding to the bar above the x-axis. The width of a bar per patient is constant. The lower panel shows the replacement pattern percentages per patient (above x-axis) and per slide (below x-axis) in the same order as in the upper panel. Note that gaps in the lower panel reflect the pushing pattern, which is not shown separately here due to its rarity. **b**) Distribution of growth patterns per slide in n = 9 patients that had progressed on chemotherapy and were operated on despite progression. The samples are sorted from left to right based on the highest replacement fraction per patient, followed by the additional sections. **c)** Comparison of growth pattern frequencies after adding n = 11 virtual patients to the analysis presented in Figure 6a. The number of patients added (n = 11) was based on historical data of operations canceled due to progression, which allowed us to estimate the expected frequency of these events (7.8% of n = 145 patients treated with chemotherapy in our series); growth pattern fractions were derived from the nine patients that had progressed on chemotherapy (b), such that the two patients with the highest proportion of replacement (leftmost in [b]) were duplicated to the sum of eleven patients. Box-and-Whisker plots with median (line), interquartile range (box), minimum and maximum values within 1.5 times the IQR from the first and third quartiles (whiskers) and individual datapoints are shown; p-values from two-sided Wilcoxon-tests are shown in the panel.

Supplementary Figure 9

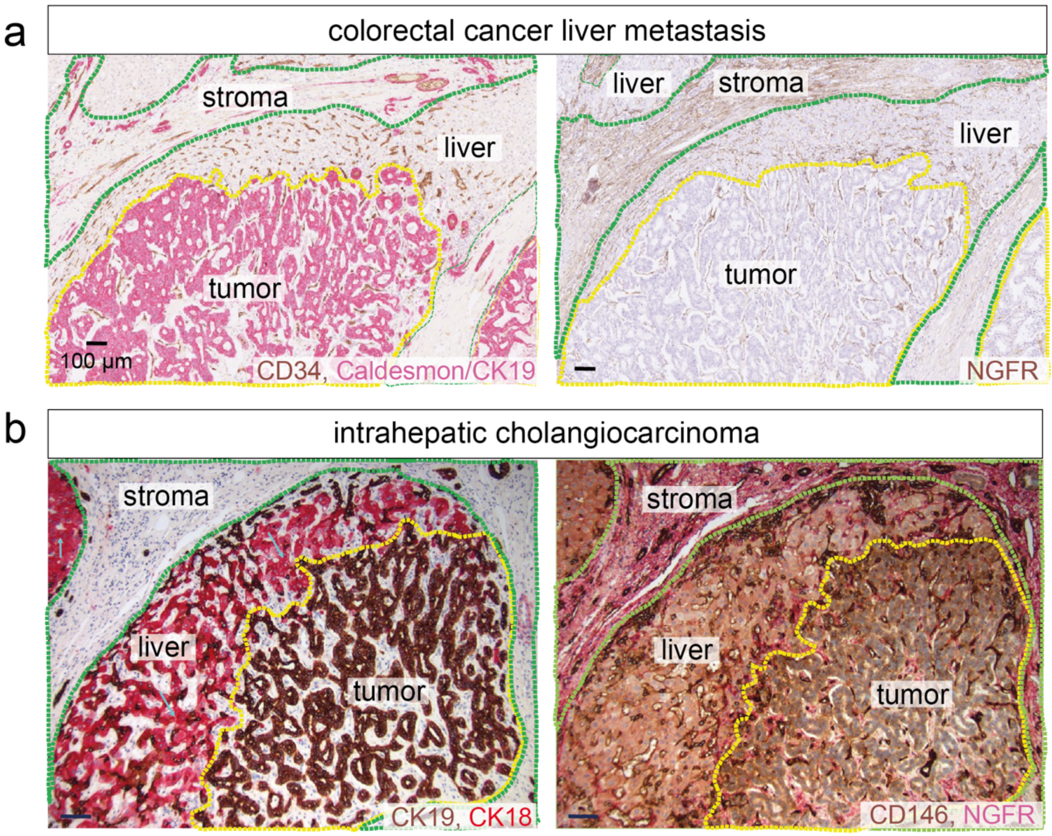

Supplementary Figure 9: Replacement growth pattern in cirrhotic liver. **a)** Metastatic colorectal cancer and **b)** intrahepatic cholangiocarcinoma (yellow dotted areas mark the tumors) invade nodules of hepatic parenchyma (labeled "liver"), thereby establishing direct contact with the hepatocytes at the invasion front (replacement-type growth). Regenerative liver nodules surrounded by fibrotic septa (green dotted areas) are definite features of liver cirrhosis. The stroma is positive for nerve growth factor receptor (NGFR), stained in brown in (a, right panel) and in red in (b, right panel). Tumor cells are positive for cytokeratin 19 (CK19), stained in red in (a, left panel) and in brown in (b, left panel). CK18 stains regenerative hepatocytes (b, left panel). (a) is representative of this individual case. (b) is representative of $n > 5$ routinely observed patients.

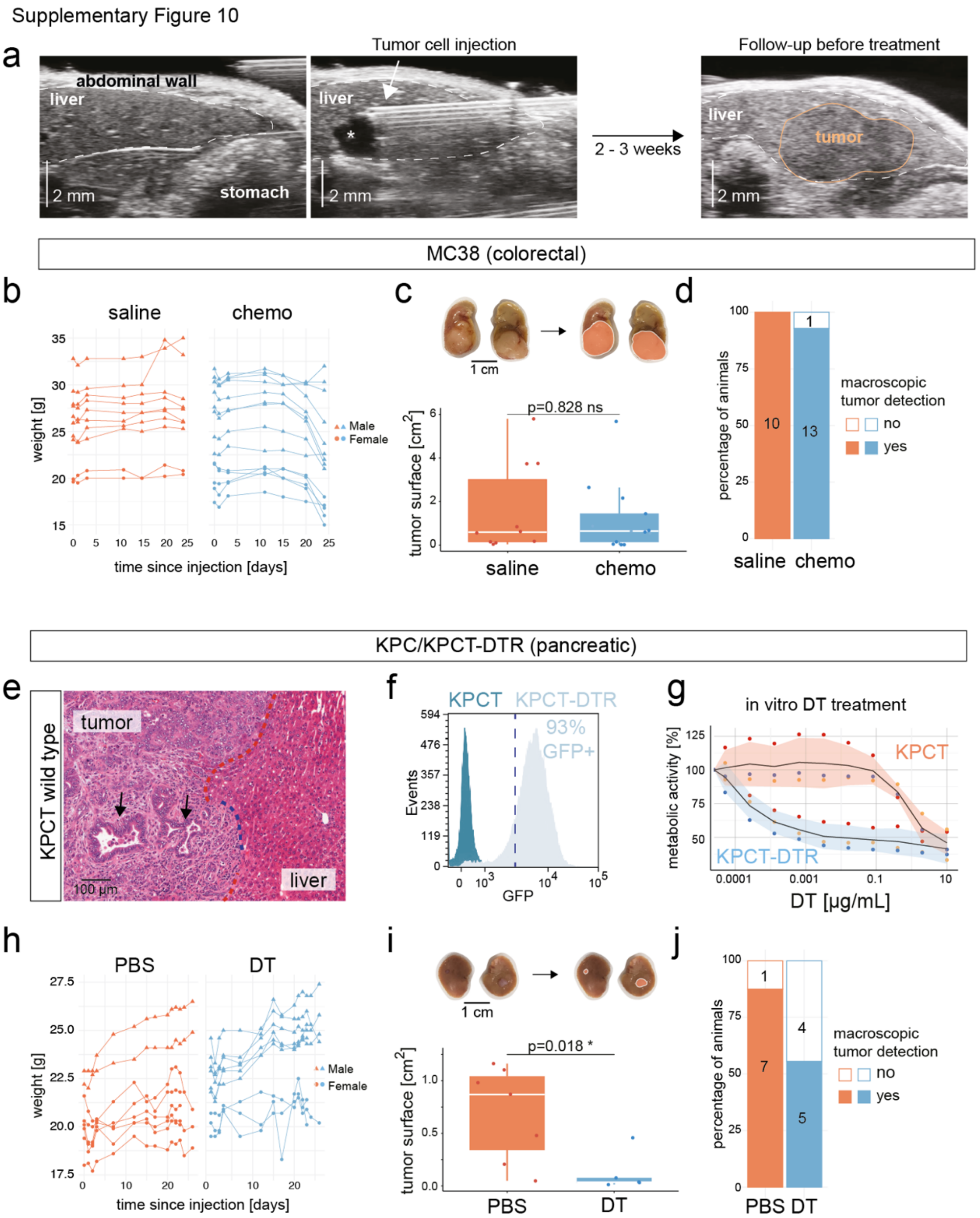

Supplementary Figure 10: Mouse metastases models. a) Metastases were induced by ultrasound-guided intrahepatic injection of a cell suspension (star) through the tip of a 30-gauge needle (arrow). Tumor development was confirmed by ultrasound on day 15. Mice in (**b-d)** were injected with colorectal cancer cells (MC38) and treated with 5-Fluorouracil and Oxaliplatin intraperitoneally on days 15 and 19. Saline was used as solvent and for volume-adjusted injection of control animals. **b**) Body weight during the treatment for each animal. **c**) The tumor surface area of macroscopically detectable lesions as quantified on post-mortem captured images as indicated. **d**) Absolute numbers of macroscopically visible tumor lesions on autopsy. **e)** Intrahepatic injection of pancreatic cancer cells ("KPCT") results in liver metastases predominantly characterized by replacement growth with features of glandular differentiation (arrows), closely resembling the histology of human metastases. Growth patterns indicated in blue (encapsulated) and red (replacement). Representative of metastases from n > 20 mice injected with KPCT cells and not otherwise treated. **f)** Result from fluorescent-activated cell sorting (FACS) analysis of KPCT-DTR cells based on the GFP encoded by the DTR construct. **g**) Results from *in vitro* assay with 3-[4,5-dimethylthiazol-2-yl]-2,5 diphenyl tetrazolium bromide (MTT) to assess cell metabolism after incubation of KPCT-DTR cells for 24h with DT. Non-transfected KPCT cells served as reference. Line shows median, areas indicate standard deviation. **h-j**) Weight curves and tumor sizes of mice treated with DT *vs*. control (phosphate buffered saline). Box-and-Whisker plots with median (line), interquartile range (box), minimum and maximum values within the 1.5 interquartile range (whiskers) and individual datapoints are shown in (c) and (i), and p-values from two-sided Wilcoxon test are shown in the panels.

Supplementary Figure 11

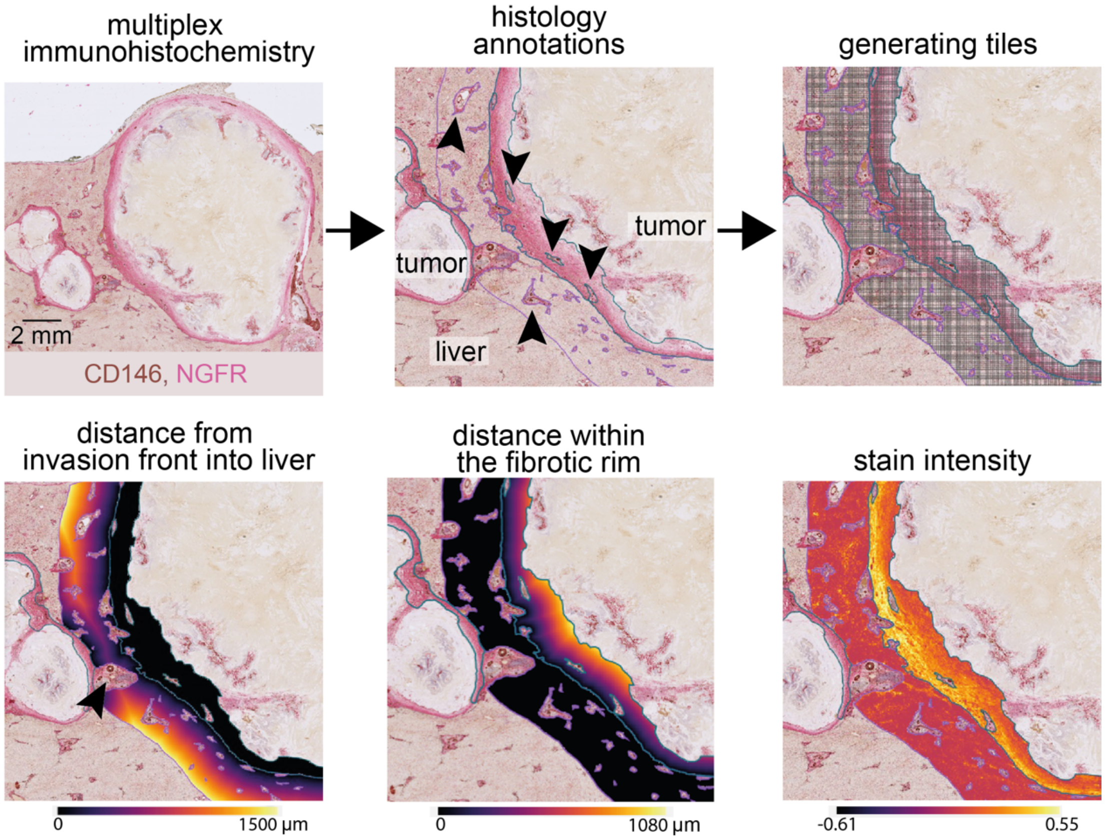

Supplementary Figure 11: Workflow to quantify immunohistochemistry (IHC) and immunofluorescense (IF) stains in QuPath. Cluster of differentiation (CD) 146 and nerve growth factor receptor (NGFR) double stain (upper left panel). First, histological structures are annotated (fibrotic rim, perimetastatic liver parenchyma, upper middle panel, corresponding to blue and purple colored lines respectively) and portal triads (arrowheads) are excluded. Tiles of size 25 µm x 25 µm are created to assign spatial location (= tile center) to stain intensity (upper right). Tile distances from the invasion front into the perimetastatic liver parenchyma (lower left) and within the rim (lower middle). NGFR stain intensity (average tile expression, lower right) after deconvolution of the stains. Each tile is then defined by its distances to annotated structures and its average marker intensity. A similar approach was used to quantify RNA *in situ* hybridization stains.

Supplementary Table 1: Comparison of patients that received chemotherapy and those that did not. Columns 1 and 2: Number of patients or median with interquartile range in brackets. Column 3: Results from Pearson's Chi-squared test, Wilcoxon rank sum test (two-sided), and Fisher's exact test (two-sided), where applicable. ASA: American Society of Anesthesiologists, WHO: World Health Organization, *KRAS*: Kirsten rat sarcoma virus gene, MSI: microsatellite instability, *BRAF*: B-Raf Proto-Oncogene.

Variable	n	Neoadjuvant chemotherapy		p-value
		No, n = 96	Yes, n = 165	
**Sex**	261			0.00442
female		26 (27%)	74 (45%)	
male		70 (73%)	91 (55%)	
**Age**	261	72 (63, 78)	66 (57, 71)	0.000000109
**ASA classification**	261			0.0000109
1		22 (23%)	76 (46%)	
2		15 (16%)	37 (22%)	
3		59 (61%)	52 (32%)	
**WHO performance status**	260			0.000000370
0		69 (72%)	156 (95%)	
1		25 (26%)	8 (4.9%)	
2		2 (2.1%)	0 (0%)	
unknown		0	1	
**Charlson comorbidity index**	260			0.00000000115
6 – 8		32 (33%)	103 (63%)	
9		26 (27%)	48 (29%)	
10 – 14		38 (40%)	13 (7.9%)	
unknown		0	1	
**Location of primary tumor**	259			0.758
left		74 (78%)	125 (76%)	
right		21 (22%)	39 (24%)	
unknown		1	1	
**Tumor stage (T)**	235			0.556
0		0 (0%)	3 (2.0%)	
1		0 (0%)	2 (1.4%)	
2		11 (13%)	20 (14%)	
3		57 (66%)	85 (57%)	
4		19 (22%)	38 (26%)	
unknown		9	17	
**Nodal stage (N)**	233			0.799
0		31 (36%)	52 (35%)	
1		36 (42%)	57 (39%)	
2		19 (22%)	38 (26%)	
unknown		10	18	
**Resected primary tumor**	259	93 (97%)	152 (93%)	0.213
unknown		0	2	
**Radicality, primary tumor**	207			0.637
R0		77 (96%)	123 (97%)	
R1		2 (2.5%)	4 (3.1%)	
R2		1 (1.3%)	0 (0%)	
unknown		16	38	
**Meta-/ synchronous metastasis**	250			0.000000915
metachronous		66 (71%)	61 (39%)	
synchronous		27 (29%)	96 (61%)	
unknown		3	8	
**Number of liver metastases**	243			0.000143
<4		85 (94%)	115 (75%)	
4+		5 (5.6%)	38 (25%)	
unknown		6	12	
**Mean number of liver metastases**	243	1 (1, 2)	2 (1, 3)	0.000000353
unknown		6	12	
**Max. diameter of individual metastasis**	261			0.0641
<5 cm		74 (77%)	142 (86%)	
5 cm		22 (23%)	23 (14%)	
**Resection margin (liver metastasis)**	231			0.532
<1 mm		38 (44%)	58 (40%)	
1 mm +		48 (56%)	87 (60%)	
unknown		10	20	
**Sum of all metastasis diameters (cm)**	261	3.0 (2.0, 6.5)	3.0 (1.4, 6.0)	0.346
**Metastases regression [%]**	232	46 (35, 60)	28 (9, 46)	0.00000000356
unknown		12	17	
**Cases with KRAS mutation**	102	4 (25%)	38 (44%)	0.152
unknown		80	79	
**MSI-high cases**	14	0 (0%)	1 (11%)	1
unknown		91	156	
**Cases with BRAF mutation**	81	1 (7.1%)	0 (0%)	0.173
unknown		82	98	

Supplementary Table 2: Histopathological features of the non-tumorous liver parenchyma in patients with predominantly replacement *vs*. encapsulated growth pattern. Patients with either >85% replacement or >85% encapsulated growth are included. Results from Pearson's Chi-squared test (two-sided).

Variable	*n*	*Predominant growth pattern*		*p-value*
		**Encapsulated, n = 77**	**Replacement, n = 43**	
Inflammation	102			0.0856
>0		43 (67%)	19 (50%)	
0		21 (33%)	19 (50%)	
unknown		13	5	
Fibrosis	116			0.209
>0		65 (87%)	39 (95%)	
0		10 (13%)	2 (4.9%)	
unknown		2	2	
Steatosis	118			0.682
>0		61 (80%)	35 (83%)	
0		15 (20%)	7 (17%)	
unknown		1	1	
Iron deposits	45			0.329
>0		8 (33%)	10 (48%)	
0		16 (67%)	11 (52%)	
unknown		53	22	

Supplementary Table 3: Antibodies used for multiplex-immunohistochemistry. *CRP stains were done using a Ventana BenchMark Ultra autostainer, all other stains as described in Methods. Antibody clones may have varied during the time span of the retrospective study due to availability for clinical use and the predominantly used clones are listed below. All new clones and antibodies are routinely validated on test sections prior to introduction to clinical routine.

Antibody	*Clone name*	*Dilution*	*Supplier name*	*Catalogue number*
Actin (smooth muscle)	1A4	1:500	Dako	M0851
Caldesmon	h-CD	1:300	Dako	M3557
CD34	QBEnd/10	1:50	Dako	M7165
CD68	PG-M1	1:100	Dako	M0876
CK18	DC-10	1:50	Dako	M7010
CK7	RN7	1:200	Leica	NCL-L-CK7-560
CD146	UMAB154	1:200	OriGene	UM800051
NGFR	polyclonal	1:500	Atlas Antibodies	HPA004765
CRP	Y284	1:200	Abcam	ab32412*
CK19	A53-B/A2.26	1:100	Sigma-Aldrich	319M-16
CK20	SP33	1:100	Abcam	ab64090
