# Supplementary material for: An idiosyncratic zonated stroma encapsulates desmoplastic liver metastases and originates from injured liver
Source: Nat Commun. 2023 Aug 18;14:5024. doi: 10.1038/s41467-023-40688-x (PMC10439160; doi:10.1038/s41467-023-40688-x)
Supplement: Supplementary file 3 — Reporting Summary [file 41467_2023_40688_MOESM3_ESM.pdf]

nature portfolio

Corresponding author(s): Marco Gerling
Last updated by author(s): Jun 19, 2023

Reporting Summary

Nature Portfolio wishes to improve the reproducibility of the work that we publish. This form provides structure for consistency and transparency in reporting. For further information on Nature Portfolio policies, see our Editorial Policies and the Editorial Policy Checklist.

Statistics

For all statistical analyses, confirm that the following items are present in the figure legend, table legend, main text, or Methods section.

n/a	Confirmed	
☐	☒	The exact sample size (*n*) for each experimental group/condition, given as a discrete number and unit of measurement
☐	☒	A statement on whether measurements were taken from distinct samples or whether the same sample was measured repeatedly
☐	☒	The statistical test(s) used AND whether they are one- or two-sided<br>*Only common tests should be described solely by name; describe more complex techniques in the Methods section.*
☐	☒	A description of all covariates tested
☐	☒	A description of any assumptions or corrections, such as tests of normality and adjustment for multiple comparisons
☐	☒	A full description of the statistical parameters including central tendency (e.g. means) or other basic estimates (e.g. regression coefficient) AND variation (e.g. standard deviation) or associated estimates of uncertainty (e.g. confidence intervals)
☐	☒	For null hypothesis testing, the test statistic (e.g. *F*, *t*, *r*) with confidence intervals, effect sizes, degrees of freedom and *P* value noted<br>*Give P values as exact values whenever suitable.*
☒	☐	For Bayesian analysis, information on the choice of priors and Markov chain Monte Carlo settings
☒	☐	For hierarchical and complex designs, identification of the appropriate level for tests and full reporting of outcomes
☐	☒	Estimates of effect sizes (e.g. Cohen's *d*, Pearson's *r*), indicating how they were calculated

Our web collection on statistics for biologists contains articles on many of the points above.

Software and code

Policy information about availability of computer code

Data collection	The custom-made scripts used to generate the plots are available at Github: https://github.com/gerlingm/CRLM_analysis<br>R version 2023.03.0+386 was used to generate the final plots. NDP.view 2 (U12388-01) was used to annotate the growth patterns. QPath version 0.2.3. was used to quantify IHC stains.<br>Other software versions are specified in the documentation where applicable (readme).
Data analysis	Please see above, "Data collection". The same software and code was used for collection and analysis of data.

For manuscripts utilizing custom algorithms or software that are central to the research but not yet described in published literature, software must be made available to editors and reviewers. We strongly encourage code deposition in a community repository (e.g. GitHub). See the Nature Portfolio guidelines for submitting code & software for further information.

Data

Policy information about availability of data

All manuscripts must include a data availability statement. This statement should provide the following information, where applicable:

- Accession codes, unique identifiers, or web links for publicly available datasets
- A description of any restrictions on data availability
- For clinical datasets or third party data, please ensure that the statement adheres to our policy

Source data are provided with this paper. Clinical data is not publicly available, in accordance with data protection regulations, as the data contain potentially

identifying or sensitive patient information. Anonymized data will be made available upon request to the corresponding author to the full extent legally possible and requests will be answered within the time frame stated in the Methods section.

Human research participants

Policy information about studies involving human research participants and Sex and Gender in Research.

Reporting on sex and gender	Sex was determined based on the clinical charts, which in most cases will reflect the biological gender at birth and in individual rare cases might reflect self-reported sex. All reports are on a group level, no individual sex is reported. Sex is given in Tables 1 and 2. Sex was used to define strata in survival analyses; there was no difference in survival between males and females (log-rank p=0.99). Neither was sex significant in univariate analysis of death and relapse. Therefore, sex was not considered further in downstream analysis.
Population characteristics	This information is given in Table 1.
Recruitment	All consecutive patients operated on liver metastases were included (2012-15) at Karolinska University Hospital, no patients were additionally recruited. Karolinska Hospital serves all of region Stockholm and adjacent counties and patients are referred to Karolinska based on geographical location. No other clinics perform a relevant number of hepatactomies for the investigated patient population, such that coverage of nearly all patients in this region and time frame can be assumed.
Ethics oversight	The Swedish National Ethical Review Board, Etikprövningsmyndigheten, gave ethical approval for all the work on human samples (#2019/01571 and #2021/06863-0, as well as #2018/1261-31 and 2019/05198); informed consent was waived. The participants received no compensation. The Swedish Board of Agriculture approved the animal experiments via the regional ethics committee, Linköpings djurförsöksetiska nämnd (#217-2022 and # 22149-2022).

Note that full information on the approval of the study protocol must also be provided in the manuscript.

Field-specific reporting

Please select the one below that is the best fit for your research. If you are not sure, read the appropriate sections before making your selection.

☒ Life sciences ☐ Behavioural & social sciences ☐ Ecological, evolutionary & environmental sciences

For a reference copy of the document with all sections, see nature.com/documents/nr-reporting-summary-flat.pdf

Life sciences study design

All studies must disclose on these points even when the disclosure is negative.

Sample size	No sample size calculations were performed for the human experiments. All consecutive patients of a predetermined time period were included. The time period (2012-15) was chosen because it allowed for long follow-up, most modern therapies, and because it was feasible to manually annotate the growth patterns for this number of patients/histological sections. The latter factor precluded building a larger cohort. An extended cohort was used in the revised manuscript, including all patients operated on at our hospital from 2012 until 2020. This was the longest available time period approved from the national ethical review board based on the decision on the permit that was available for this revision.<br><br>For mouse experiments, prior estimation of the sample size was done using pnorm in R, assuming a mean of 95% (standard deviation 5%) replacement in vehicle-treated mice and a mean of 80% replacement (standard deviation 15%) in treated mice (DT or chemotherapy) and a sampling ratio of 1, yielding a sample size of n = 7 with a power of 0.8 and a type 1 error rate of 5%. In total for each experiment, n = 30 mice were included for tumor injection to account for tumor engraftment failure and errors in the assumptions.
Data exclusions	Patients were excluded when the growth pattern could not be determined (in case of a complete regression), or when no representative histological sections could be retrieved. This is specified in the Results.
Replication	Annotations were done by 2 physicians (annotator/supervisor). The first 94 patients were assessed by the world leading expert in the field of liver metastases growth patterns. Cohen's Kappa was calculated as described in the manuscript.<br>Investigators were blinded to clinical outcome (human data) or treatment groups (murine data).<br>Growth pattern annotations were done by two investigators, such that the annotations were first done by one investigator and subsequently improved/corrected by a specialist pathologist (CFM). Murine experiments were done once with the indicated numbers of animals.
Randomization	Does not apply to human data. The mouse experiments were randomized as specified in Methods.<br>The analyses of the human data were retrospectively and inherently non-randomized. Covariates were controlled for in multivariate analysis (Figure 2).
Blinding	All growth patterns annotations were done blinded to clinical outcome/treatment groups of mouse experiments. Clinical charts were reviewed blinded to the growth patterns. Growth patterns of murine tumors were assessed blinded to the treatment groups. In vitro experiments (cell culture and treatment with diphteria toxin) were not blinded as they were done by one single investigator (NG).

Reporting for specific materials, systems and methods

We require information from authors about some types of materials, experimental systems and methods used in many studies. Here, indicate whether each material, system or method listed is relevant to your study. If you are not sure if a list item applies to your research, read the appropriate section before selecting a response.

Materials & experimental systems

n/a	Involved in the study
☐	☒ Antibodies
☒	☐ Eukaryotic cell lines
☒	☐ Palaeontology and archaeology
☐	☒ Animals and other organisms
☒	☐ Clinical data
☒	☐ Dual use research of concern

Methods

n/a	Involved in the study
☒	☐ ChIP-seq
☒	☐ Flow cytometry
☒	☐ MRI-based neuroimaging

Antibodies

Antibodies used

Clinical antibodies are given in Supplementary Table 3. Antibodies used for multiplex immunofluorescence are listed in Methods. All antibodies are given below together with their validation statements ("Validation").

Validation

Validation statements are listed below:

Anti-LoxL2: Cell Signaling Technology #99680 rabbit monoclonal clone E3P7Y Lot:1
Recommended by manufacturer for IHC on FFPE; antibody guarantee provided by the manufacturer and product is stated to specifically recognize endogenous levels of total LOXL2 protein, however IHC validation data not shown on the manufacturer homepage (https://www.cellsignal.com/products/primary-antibodies/loxl2-e3p7y-rabbit-mab/99680?_=1686999822390&Ntt=E3P7Y&tahead=true), specificity demonstrated via Western Blot with positive protein band detection for LoxL2 positive cell line BT-549, while no band was detected for LoxL2 negative cell line HCT-15.
No additional validation assays were performed.

Anti-ASMA: Agilent M085101-2 mouse monoclonal clone 1A4 concentrate Lot: 20039209
Recommended by manufacturer for IHC on FFPE, antibody is certified for in vitro diagnostic use (CE-IVD). No IHC validation data provided by manufacturer, but the manufacturer states that IHC stained normal tissue samples show cytoplasmatic staining pattern for expected cell types such as e.g. smooth muscle cells, myoepithelial cells (https://www.agilent.com/cs/library/packageinsert/public/SSM0851CEEFG_02.pdf).
No additional validation assays were performed.

Anti-PDGFRa: Cell Signaling Technology #5241 rabbit monoclonal clone D13C6 Lot: 4
Cross-reactivity with PDGFRb excluded in IHC tests on FFPE embedded porcine aortic endothelial cells transfected to express either human PDGFRa or PDGFRb protein (Strell et al, JNCI 2019 Sept;111(9):983-995 Supplementary Fig 1A).
Recommended by manufacturer for IHC on FFPE; antibody guarantee provided by the manufacturer and product is stated to specifically recognize endogenous levels of PDGFRa protein; IHC validation data on the manufacturer homepage shows staining for PDGFRa on FFPE embedded PDGFRa-positive NCI-H1703 cells while no staining detected for PDGFRa-negative HCC827 cells (https://www.cellsignal.com/products/primary-antibodies/pdgf-receptor-a-d13c6-xp-rabbit-mab/5241?_=1687000040824&Ntt=D13C6&tahead=true)

Anti-PDGFRb: Cell Signaling Technology #3169 rabbit monoclonal clone 28E1 Lot: 13
Cross-reactivity with PDGFRb excluded in IHC tests on FFPE embedded porcine aortic endothelial cells transfected to express either human PDGFRa or PDGFRb protein (Strell et al, JNCI 2019 Sept;111(9):983-995 Supplementary Fig 1A; Paulsson et al AmJPathol 2009 Jul;175(1):334-41 Supplementary Fig1).
Recommended by manufacturer for IHC on FFPE; antibody guarantee provided by the manufacturer and product is stated to specifically recognize endogenous levels of PDGFRb protein, a risk for cross-reactivity with PDGFRa when highly overexpressed is noted; however, validation data not shown on the manufacturer homepage https://www.cellsignal.com/products/primary-antibodies/pdgf-receptor-b-28e1-rabbit-mab/3169?site-search-type=Products&N=4294956287&Ntt=28e1&fromPage=plp.

Anti-FAP: Cell Signaling Technology #66562 rabbit monoclonal clone E1V9V Lot: 1
Recommended by manufacturer for immunofluorescence/immunocytochemistry, but 6 references stated also successful application of the antibody in IHC; antibody guarantee provided by the manufacturer and product is stated to specifically recognize endogenous levels of total FAP protein, however validation data not shown on the manufacturer homepage (https://www.cellsignal.com/products/primary-antibodies/fap-e1v9v-rabbit-mab/66562?_=1687001840957&Ntt=E1V9&tahead=true).
No additional validation assays were performed.

Anti-phosphoSTAT3: Cell Signaling Technology #9145 rabbit monoclonal clone D3A7 Lot: 34
Recommended by manufacturer for IHC on FFPE; antibody guarantee provided by the manufacturer and product is stated to specifically recognize endogenous levels of Stat3 only when phosphorylated at tyrosine 705 without cross-reaction towards phospho-EGFR or the corresponding phospho-tyrosines of other Stat proteins; validation data for IHC shown on the manufacturer homepage

include positive nuclear staining on FFPE embedded HeLa cells stimulated with IFNa while no staining is detected on non-stimulated control HeLa cells; further control experiments demonstrate absence of positive staining on FFPE tissue sections from human breast carcinoma upon treatment of the section with lambda phosphatase, the non-treated control tissue section showed positive nuclear staining; antibody homepage: https://www.cellsignal.com/products/primary-antibodies/phospho-stat3-tyr705-d3a7-xp-rabbit-mab/9145?_=1687000910835&Ntt=D3A&tahead=true
No additional validation assays were performed.

Anti-TF: Atlas Antibodies #HPA049292 rabbit polyclonal
Recommended by the manufacturer for IF on FFPE but no IF validation data shown; 1 reference on manufacturer homepage for IHC on FFPE tissue from embryonic colon tissue; antibody homepage: https://www.sigmaaldrich.com/SE/en/product/sigma/hpa049292
No additional validation assays were performed.

Anti-alphaSMA: Abcam #AB5694 rabbit polyclonal
Recommended by the manufacturer for IHC but not IF on FFPE; antibody guarantee provided by the manufacturer; validation data for IHC shown on the manufacturer homepage include positive cytoplasmatic staining of stromal cells and negative epithelium in mouse intestine; own IF staining on mouse intestine showed the same staining pattern; antibody homepage: https://www.abcam.com/products/primary-antibodies/alpha-smooth-muscle-actin-antibody-ab5694.html?productWallTab=ShowAll
No additional validation assays were performed.

Anti-SMA: Dako #M0851 mouse monoclonal clone 1A4
Recommended by manufacturer for IHC on FFPE; antibody guarantee provided by the manufacturer; validation data for IHC shown on the manufacturer homepage include positive cytoplasmatic staining of stromal cells and negative epithel on human colon; antibody homepage: https://www.agilent.com/en/product/immunohistochemistry/antibodies-controls/primary-antibodies/actin-(smooth-muscle)-(concentrate)-76542#specifications
This antibody is extensively used in clinical routine at the Department of Pathology, Karolinska University Hospital, Huddinge, Sweden. The stains that were analyzed in this study were clinical routine stains used for diagnostics. Controls, i.e. suitable varying tissues with known expression or known absence of expression, are used on-slide on some but not all sections in clinical routine.

Anti-Caldesmon: Dako #M3557 mouse monoclonal clone h-CD
Product no longer available by manufacturer; antibody registry shows 6 references where antibody is used for IHC on FFPE (https://www.labome.com/product/Dako/M3557.html); no antibody homepage from manufacturer available.
This antibody was extensively used in clinical routine at the Department of Pathology, Karolinska University Hospital, Huddinge, Sweden. The stains that were analyzed in this study were clinical routine stains used for diagnostics; stained sections were derived from the archive, which explains that this antibody is no longer available for purchase.

Anti-CD34: Dako #M7165 mouse monoclonal clone QBEnd/10 concentrate
Recommended by manufacturer for IHC on FFPE; antibody guarantee provided by the manufacturer; immunogen were endothelial cell membranes obtained as vesicles from human placenta; validation data for IHC shown on the manufacturer homepage include positive endothelial staining in human liver sinusoids; own IHC staining on human liver metastases samples show the same staining pattern in areas with distant liver tissue (see also Figure 4f); antibody homepage: https://www.agilent.com/en/product/immunohistochemistry/antibodies-controls/primary-antibodies/cd34-class-ii-(concentrate)-76634
This antibody is extensively used in clinical routine at the Department of Pathology, Karolinska University Hospital, Huddinge, Sweden. The stains that were analyzed in this study were clinical routine stains used for diagnostics. Controls, i.e. suitable varying tissues with known expression or known absence of expression, are used on-slide on some but not all sections in clinical routine.

Anti-CD68: Dako #M0876 mouse monoclonal clone PG-M1 concentrate
Recommended by manufacturer for IHC on FFPE; antibody guarantee provided by the manufacturer; manufacturer states that clone PG-M1, in contrast to other anti-CD68 antibodies, does not stain myeloid cells but macrophages only; validation data for IHC shown on the manufacturer homepage indicate cytoplasmatic staining; own IHC staining on human liver metastases samples shows cytoplasmatic staining of cells located in the hepatic sinusoids in tissue areas with distant liver as expected for liver macrophages (see also Figure 4c); antibody homepage: https://www.agilent.com/en/product/immunohistochemistry/antibodies-controls/primary-antibodies/cd68-(concentrate)-76550
This antibody is extensively used in clinical routine at the Department of Pathology, Karolinska University Hospital, Huddinge, Sweden. The stains that were analyzed in this study were clinical routine stains used for diagnostics. Controls, i.e. suitable varying tissues with known expression or known absence of expression, are used on-slide on some but not all sections in clinical routine.

Anti-CK18: Dako #M7010 mouse monoclonal clone DC-10 concentrate
Recommended by manufacturer for IHC on FFPE; antibody guarantee provided by the manufacturer; validation data for IHC shown on the manufacturer homepage indicate strong cytoplasmatic staining in epithelial tumors as well as bile duct epithelium in the liver and weak to moderate membranous and cytoplasmatic staining in the remaining liver epithelium; own IHC staining on human liver metastases samples shows the same staining pattern in distant liver (see also Figure 4c); antibody homepage: https://www.agilent.com/en/product/immunohistochemistry/antibodies-controls/primary-antibodies/cytokeratin-18-(concentrate)-76619
This antibody is extensively used in clinical routine at the Department of Pathology, Karolinska University Hospital, Huddinge, Sweden. The stains that were analyzed in this study were clinical routine stains used for diagnostics. Controls, i.e. suitable varying tissues with known expression or known absence of expression, are used on-slide on some but not all sections in clinical routine.

Anti-CD146: OriGene #UM800051 mouse monoclonal clone UMAB154
Recommended by the manufacturer for IHC on FFPE; validation data for IHC shown for human melanoma, endometrium adenocarcinoma, colon adenocarcinoma, pancreas carcinoma, thyroid carcinoma, liver carcinoma, lung carcinoma, lymph node, endometrium, pancreas, prostate, lymphoma, kidney, ovary, tonsil tissue on the manufacturer homepage; antibody homepage: https://www.thermofisher.com/antibody/product/MCAM-Antibody-clone-UMAB154-Monoclonal/UM800051
This antibody is extensively used in clinical routine at the Department of Pathology, Karolinska University Hospital, Huddinge, Sweden. The stains that were analyzed in this study were clinical routine stains used for diagnostics. Controls, i.e. suitable varying tissues with known expression or known absence of expression, are used on-slide on some but not all sections in clinical routine.

Anti-NGFR: Atlas Antibodies #HPA004765 rabbit polyclonal
Recommended by manufacturer for IHC on FFPE; Antibody homepage: https://www.sigmaaldrich.com/SE/en/product/sigma/hpa004765
This antibody is extensively used in clinical routine at the Department of Pathology, Karolinska University Hospital, Huddinge, Sweden. The stains that were analyzed in this study were clinical routine stains used for diagnostics. Controls, i.e. suitable varying tissues with known expression or known absence of expression, are used on-slide on some but not all sections in clinical routine.
Anti-CRP: Abcam #ab32412 rabbit monoclonal clone Y284
Recommended by the manufacturer for IHC on FFPE; antibody guarantee provided by the manufacturer; validation data for IHC shown on the manufacturer homepage indicate cytoplasmatic staining in the liver epithelial cells; own IHC shows similar staining pattern (see also Figure 4b); antibody homepage: https://www.abcam.com/products/primary-antibodies/c-reactive-protein-antibody-y284-ab32412.html
This antibody is extensively used in clinical routine at the Department of Pathology, Karolinska University Hospital, Huddinge, Sweden. The stains that were analyzed in this study were clinical routine stains used for diagnostics. Controls, i.e. suitable varying tissues with known expression or known absence of expression, are used on-slide on some but not all sections in clinical routine.

Anti-CK7: Leica #NCL-L-CK7-560 mouse monoclonal clone RN7
Recommended by the manufacturer for IHC on FFPE; manufacturer validated antibody on a large number of normal epithelial and cancer cells, among them liver bile ducts and described cytoplasmatic and membranous staining pattern in epithelial cells; positive liver bile duct staining served as internal validation control (see also Figure 4e); antibody homepage: https://shop.leicabiosystems.com/de/pid-CK7-560-L-CE
This antibody is extensively used in clinical routine at the Department of Pathology, Karolinska University Hospital, Huddinge, Sweden. The stains that were analyzed in this study were clinical routine stains used for diagnostics. Controls, i.e. suitable varying tissues with known expression or known absence of expression, are used on-slide on some but not all sections in clinical routine.
Anti-CK19: Sigma-Aldrich #319M-16 mouse monoclonal clone A53-B/A2.26 concentrate

Recommended by the manufacturer for IHC on FFPE; antibody guarantee provided by the manufacturer; validation data for IHC shown on the manufacturer homepage indicate cytoplasmatic staining of epithelial tumor cells; own IHC show staining in intrahepatic cholangiocarcinoma cells but no staining in stroma or liver epithel (Supplementary Figure 9b); antibody homepage: https://www.sigmaaldrich.com/SE/en/product/sigma/319m1
This antibody is extensively used in clinical routine at the Department of Pathology, Karolinska University Hospital, Huddinge, Sweden. The stains that were analyzed in this study were clinical routine stains used for diagnostics. Controls, i.e. suitable varying tissues with known expression or known absence of expression, are used on-slide on some but not all sections in clinical routine.

Anti-CK20: Abcam #ab64090 rabbit monoclonal clone SP33
Recommended by manufacturer for IHC on FFPE; validation data for IHC shown on the manufacturer homepage indicate moderate to strong cytoplasmatic and membranous staining of human colon carcinoma cells; own IHC show similar staining pattern in human colon carcinoma liver metastases (Figure 4e); antibody homepage: https://www.abcam.com/products/primary-antibodies/cytokeratin-20-antibody-sp33-ab64090.html#lb
This antibody is extensively used in clinical routine at the Department of Pathology, Karolinska University Hospital, Huddinge, Sweden. The stains that were analyzed in this study were clinical routine stains used for diagnostics. Controls, i.e. suitable varying tissues with known expression or known absence of expression, are used on-slide on some but not all sections in clinical routine.

Anti-mouse IgG: Vector Laboratories ImmPress-mouse HRP #MP-7402 horse polyclonal, conjugated with horseradish peroxidase
Recommended by manufacturer for IHC on FFPE; on the manufacturers homepage there are currently 249 references linked to the antibody; antibody homepage: https://vectorlabs.com/products/enzyme-polymer/immpress-hrp-horse-anti-mouse-igg-kit#description
No additional validation assays were performed.

Anti-rabbit IgG: Vector Laboratories ImmPress-mouse HRP #MP-7401 horse polyclonal, conjugated with horseradish peroxidase
Recommended by manufacturer for IHC on FFPE; manufacturer provides positive validation results for the antibody on 3 automated IHC platforms in combination with a von Willebrand factor primary antibody on human skeletal muscle FFPE sections; on the manufacturers homepage there are currently 711 references linked to the antibody; antibody homepage: https://vectorlabs.com/products/enzyme-polymer/immpress-hrp-horse-anti-mouse-igg-kit#description
No additional validation assays were performed.

Anti-rabbit IgG: Thermo Fisher Scientific #A21245 goat polyclonal secondary antibody, conjugated with Alexa Fluor 647
Recommended by manufacturer for IF on FFPE; to reduce cross-reactivity the antibody has been absorbed against bovine, goat, mouse, rat, and human IgG by the manufacturer; on the manufacturers homepage there are currently 1526 references linked to the antibody; antibody homepage: https://www.thermofisher.com/antibody/product/Goat-anti-Rabbit-IgG-H-L-Highly-Cross-Adsorbed-Secondary-Antibody-Polyclonal/A-21245
No additional validation assays were performed.

Animals and other research organisms

Policy information about studies involving animals; ARRIVE guidelines recommended for reporting animal research, and Sex and Gender in Research

Laboratory animals	C57BL/6J mice obtained from Charles River were used for all experiments. Female and male mice at 9-12 weeks of age were included. Mice were housed in specific-pathogen-free conditions at a 12h light/dark cycle at circa 20 – 22 °C and fed standard chow.<br><br>KPCT cells were derived from KPCT mice in earlier studies. The strains to generate KPC-T cells were: KrasLSL-G12D/+;Trp53LSL-R172H/+;Pdx-Cre (Hingorani, Cell, 2005, PMID 15894267); B6.Cg-Gt(ROSA)26Sortm9(CAG-tdTomato)Hze/J.

Wild animals	no wild animals were used
Reporting on sex	The sex of each animal is reported in the Source Data. Both male and female mice were used and sex was not considered for randomization. The small sample sizes do not allow for meaningful disaggregation of the data.
Field-collected samples	no field-collected samples were used.
Ethics oversight	The Swedish Board of Agriculture approved the animal experiments via the regional ethics committee, Linköpings djurförsöksetiska nämnd (#217-2022 and # 22149-2022) and this is stated in Methods.

Note that full information on the approval of the study protocol must also be provided in the manuscript.
